# Experimental Development of an Enriched Tomato Juice with Bioactive Extracts from Unripe Green Tomatoes

**DOI:** 10.3390/molecules31132210

**Published:** 2026-06-23

**Authors:** Gerardina Galdi, Emanuel Mauro, Mariateresa Rapacciuolo, Maria Ilenia Sessa, Giusi Varasano, Luca Sandei

**Affiliations:** 1 SSICA (Stazione Sperimentale per l’Industria delle Conserve Alimentari) Research Foundation, Via Nazionale 121/123, 84012 Angri, SA, Italy; gerardina.galdi@ssica.it (G.G.); mauro.emanuel.93@gmail.com (E.M.); mariateresa.rapacciuolo@ssica.it (M.R.); mariailenia.sessa@ssica.it (M.I.S.); giusi.varasano@ssica.it (G.V.); 2 SSICA (Stazione Sperimentale per l’Industria delle Conserve Alimentari) Research Foundation, Viale F. Tanara 31/A, 43100 Parma, PR, Italy

**Keywords:** enriched tomato juice, bioactive extract from unripe green tomatoes, fortified food

## Abstract

The growing prevalence of chronic degenerative diseases has increased interest in nutritional strategies based on natural bioactive compounds such as polyphenols. This study aimed to develop a polyphenol-fortified tomato juice using extracts from unripe green tomatoes and to evaluate its physicochemical, antioxidant, sensory, and storage properties. Polyphenolic extracts obtained from tomato by-products were characterized using spectrophotometric and HPLC analyses and incorporated into tomato juice, which was then pasteurized and stored for six months. Total polyphenol content increased from 40.97 to 82.45 mg GAE/100 g, decreasing to 71.44 mg after storage; HPLC confirmed higher levels of key phenolic compounds in fortified juice. DPPH antioxidant activity increased in fortified juice compared to control, since pasteurization had limited effects but decreased after storage, with a moderate reduction in bioactivity. Colorimetric and sensory analyses showed changes in color, aroma, and sweetness after storage, potentially affecting consumer acceptance, although overall composition remained largely stable. Overall, results demonstrate the feasibility of producing a polyphenol-enriched tomato juice from agro-industrial by-products with improved antioxidant properties and acceptable technological stability. These findings support the valorization of tomato processing waste and suggest potential applications in functional food development, human health promotion, and the sustainability of agri-food systems’ overall approach.

## 1. Introduction

In recent years, increasing attention has been directed toward enriched and fortified foods, driven by growing consumer awareness of the relationship between diet and health [1,2,3,4,5]. The development of enriched foods generally involves the addition of nutrients or bioactive ingredients, such as polyphenols, carotenoids, vitamins, minerals, peptides, and unsaturated fatty acids, to conventional food products in order to improve their nutritional profile and enhance their potential health benefits [2,3,5]. These bioactive compounds can be derived from a variety of natural sources, including plants, animals, and marine organisms, and are associated with a broad range of biological activities, including antioxidant, anti-inflammatory, antifungal, and antimicrobial effects [3,4,6].

Alongside the increasing demand for nutritionally enhanced foods, a substantial shift in dietary habits has been observed, characterized by a growing preference for plant-based products. This trend is largely driven by consumer demand for foods perceived as healthier and more environmentally sustainable [6]. Plant-derived ingredients represent valuable sources of bioactive compounds that can be used to enrich conventional foods and beverages, thereby increasing their nutritional value [7,8]. Evidence from preclinical and nutritional studies suggests that the regular intake of foods rich in plant-derived bioactive compounds may contribute to reducing the risk of several chronic diseases, including diabetes, cancer, cardiovascular diseases, hyperlipidaemia, and hyperuricemia [4,6,8].

Among plant-based products, tomatoes and tomato-derived products are widely recognized for their high content of bioactive compounds, including carotenoids, polyphenols, vitamins, and phytosterols, which are associated with antioxidant activity and potential health-promoting effects [9,10,11,12,13,14]. For these reasons, tomato (*Solanum lycopersicum* L.) is certainly one of the most cultivated and consumed vegetables worldwide on a global scale. Global annual production of fresh tomatoes is estimated at approximately 160 million tons, with nearly one-quarter processed industrially, making tomato the leading vegetable crop for processing industries [15].

As a consequence of this large-scale production, the tomato agri-food sector generates significant quantities of by-products, including peels, seeds, and unripe fruits, accounting for up to 0.5–3% of total production [16,17]. These residues are commonly used for the production of sauces, snacks, and animal feed; however, they also represent a valuable source of bioactive compounds, including phenolics, carotenoids, tocopherols, and glycoalkaloids [17,18]. Increasing scientific interest has therefore been directed toward their valorization as functional ingredients for food and nutraceutical applications [17].

The main compositional differences between green and ripe tomatoes are related to secondary metabolites, including polyphenols, carotenoids, ascorbic acid, and chlorophylls. In particular, polyphenol content varies significantly between ripening stages, as these compounds are involved in plant defense mechanisms and tend to decrease or remain stable during maturation [10,19]. Consequently, fruit ripening should not be considered merely a process of compositional enrichment, but rather a profound reprogramming of secondary metabolism. Unripe green tomatoes, therefore, represent a particularly rich source of antioxidant and bioactive compounds and should be regarded as a valuable raw material rather than an agricultural by-product [17,19,20,21,22].

The recovery and valorization of bioactive compounds from agro-industrial by-products have gained considerable interest as sustainable strategies for the development of enriched foods and for reducing food waste [23]. Although previous studies have investigated tomato-based enriched beverages, polyphenol fortification strategies, and extraction of bioactive compounds from tomato residues, limited attention has been paid to the direct use of extracts obtained from unripe green tomatoes in beverage formulation [16,17].

The utilization of unripe tomatoes thus represents a promising strategy to enhance the nutritional and antioxidant properties of tomato-based beverages, while supporting circular economy approaches in the agri-food sector [16].

Accordingly, the aim of the present study was to develop and characterize a novel tomato juice enriched with natural extracts obtained from unripe green tomatoes, evaluating the effects of enrichment on physicochemical properties, bioactive compound content, antioxidant capacity, and sensory acceptability, with the objective of proposing an innovative and sustainable enriched tomato-based beverage with improved nutritional value.

## 2. Results and Discussion

The polyphenolic extract used to fortify the tomato juice has been obtained directly from unripe green tomatoes through a semi-industrial prototype system, located at the Technology Laboratory of the SSICA Research Foundation—Stazione Sperimentale per l’Industria delle Conserve Alimentari, sited in Angri (SA). This experimental cascade processing line consisted of a conventional tomato juice production plant unit, integrated with an ultrasound (US) pre-treatment step combined with a complementary accelerated solvent extraction (ASE) unit operating under high pressure. The experimental plant system enabled controlled processing conditions that better preserved the functional properties of the polyphenolic fraction while ensuring product stability and process scalability [24].

Moreover, the natural extract obtained has been added to a red tomato juice immediately prior to packaging in glass jars and subsequent thermal pasteurization, and then stored at room temperature. The amount of extract added was determined through preliminary fortification trials with the aim of doubling the polyphenol content.

The analyses have been carried out on the red tomato juice, on the fortified juice and on the fortified juice after six months of storage.

### 2.1. Total Polyphenols

The analysis revealed a significant increase in total polyphenol content in the fortified tomato juice compared to the control tomato juice, indicating the effectiveness of enrichment with the polyphenolic extract obtained from unripe green tomatoes (Table 1). Although the extract itself exhibited a much higher phenolic concentration (1788.89 mg GAE 100 g^−1^), this value refers to the concentrated crude extract prior to incorporation and is not directly comparable with the final product due to dilution effects during formulation.

After 6 months of storage, a slight decrease in total polyphenol content was observed. Overall, the retention of total polyphenols during storage was approximately 86.6%, indicating good stability of phenolic compounds under the applied storage conditions.

From a technological standpoint, the observed decrease can be mainly attributed to oxidative degradation processes occurring during storage, potentially promoted by residual oxygen and light exposure, as well as to possible interactions with the food matrix, such as binding to proteins and polysaccharides or complexation phenomena, which may reduce the extractable fraction and thus the measured content. Nevertheless, the overall reduction remained limited and technologically acceptable, as a substantial proportion of bioactive compounds was preserved over time, suggesting that the thermal treatment and storage conditions were adequate to maintain the functional quality of the fortified juice throughout its shelf life.

### 2.2. DPPH Radical Scavenging Activity

The DPPH assay showed that fortification significantly increased the antioxidant activity of tomato juice compared with the control (Table 2). Immediately after production, the fortified sample exhibited higher radical scavenging activity in both methanolic (46.08%) and acetonic extracts (45.41%) compared with non-fortified tomato juice (37.85% and 23.02%, respectively), confirming the effective contribution of the added polyphenolic extract.

The differences observed between the two extraction systems can be attributed to the different polarities of the solvents used. In particular, methanol, being a more polar solvent than acetone, is more efficient in extracting highly polar phenolic compounds, whereas acetone shows greater affinity for less polar or more lipophilic phenolic molecules. Therefore, solvent type may significantly influence the selectivity of phenolic compound extraction and, consequently, the measured antioxidant activity [25].

After six months of storage, a decrease in antioxidant activity was observed in both extraction systems. The methanolic extract decreased to 35.50%, while the acetonic extract decreased to 34.02%. Although a reduction was evident, the fortified juice still maintained antioxidant activity values comparable to or higher than the control.

The observed decline during storage may be attributed to the partial degradation of antioxidant compounds, as well as possible interactions with the food matrix that reduce their extractability and radical scavenging efficiency. Oxidative processes during storage may also contribute to the formation of derivatives with lower antioxidant capacity. It is also well established that DPPH-measured antioxidant activity is strongly dependent on the extraction solvent, as different solvent systems selectively extract distinct classes of phenolic compounds, thereby influencing the observed values.

Overall, these results indicate that fortification effectively enhances the antioxidant potential of tomato juice, although storage time leads to a partial, but not complete, reduction in activity.

### 2.3. Analysis of Phenolic Compounds in the Processed Tomato Juices 

The fortified tomato juice was mainly enriched in polyphenolic compounds such as kaempferol, naringenin, caffeic acid, p-coumaric acid, ferulic acid, and rutin. These compounds are widely recognized as plant-derived phenolics with antioxidant properties and have been extensively investigated in the literature for their potential biological activities. However, the presence of these compounds in the fortified juice does not necessarily imply comparable physiological effects in the final product, since their bioavailability, stability, and biological activity may be influenced by processing conditions and interactions with the food matrix.

Table 3 reports the polyphenolic profile of tomato juice, fortified tomato juice, and fortified tomato juice after 6 months of storage. Overall, fortification resulted in increased concentrations of several phenolic compounds, including caffeic acid, p-coumaric acid, ferulic acid, kaempferol, naringenin, and rutin. In most cases, these compounds remained relatively stable during storage, suggesting that pasteurization and the applied storage conditions did not substantially affect their retention. Similar observations have been reported for several phenolic compounds subjected to moderate thermal processing, where matrix-dependent effects may partially compensate for degradation phenomena through increased extractability [26,27,28].

Nevertheless, the observed variations should be interpreted cautiously, since multiple factors may contribute to changes in polyphenol concentration following fortification and processing. Food processing is known to affect the content, extractability, and bioavailability of phenolic compounds depending on treatment intensity, duration, and matrix characteristics [27]. In particular, the comparison of kaempferol and naringenin levels between the control and fortified samples is not straightforward, as thermal treatment and storage may promote hydrolysis of glycosylated forms and conversion between bound and free phenolics, thereby altering the proportion of detectable aglycones [27,28]. This interpretation is supported by studies showing that flavonoids in tomato tissues occur predominantly as glycosylated derivatives and may be released during processing through hydrolytic reactions [29].

In contrast, chlorogenic acid and epicatechin showed lower concentrations in the fortified samples. This reduction may be partially explained by a dilution effect associated with the incorporation of the fortifying ingredient, which could modify the relative abundance of endogenous tomato polyphenols. Moreover, both compounds are known to be susceptible to oxidation and thermal degradation during processing [27,28]. In particular, epicatechin and related flavan-3-ols may undergo significant degradation depending on processing conditions and matrix composition [30]. Possible interactions with proteins, polysaccharides, dietary fiber, or other matrix constituents introduced during fortification may also have affected their extractability and analytical recovery, since polyphenol–matrix interactions are recognized as important determinants of phenolic bioaccessibility and analytical recovery [27].

Catechin and resveratrol were not detected in the fortified samples. Although degradation during processing and storage cannot be excluded, other mechanisms may also account for their absence. These compounds may have undergone oxidation, polymerization, or binding reactions with matrix components, resulting in concentrations below the analytical detection limit [27,28]. In addition, matrix-related changes in extraction efficiency may have contributed to the reduced recovery of these analytes. Similar transformation pathways have been described for phenolic compounds exposed to thermal treatment and prolonged storage, leading to the formation of degradation products or polymerized structures that are less readily detected by conventional analytical methods [28].

Ferulic acid exhibited a marked decrease after 6 months of storage, indicating lower stability over time compared with the other monitored phenolics. This observation is consistent with previous reports describing the susceptibility of ferulic acid and related hydroxycinnamic acids to oxidative and thermal degradation during prolonged storage [31,32]. Degradation products derived from ferulic acid have been reported under both thermal and storage conditions, supporting the hypothesis that chemical instability contributed to the reduction observed in the present study [32,33].

Overall, the results indicate that fortification and processing affected individual polyphenols differently, depending on their chemical structure, stability, and interactions with the food matrix. Therefore, the observed variations are likely attributable to the combined effects of formulation, thermal treatment, storage conditions, matrix interactions, and analytical recovery rather than to a single mechanism [27,28].

### 2.4. Vitamin C

The determination of vitamin C showed a marked decrease in ascorbic acid content in the fortified tomato juice compared with the control sample (Table 4), decreasing from 61.01 to 32.12 mg/100 g. This reduction can be mainly attributed to thermal degradation occurring during juice pasteurization, which is known to promote the oxidation and breakdown of ascorbic acid under heat treatment [34]. Therefore, the results indicate that the fortification with the polyphenolic extract did not prevent vitamin C losses associated with thermal processing.

### 2.5. Carotenoids

The determination of lycopene content, the main carotenoid in tomatoes, showed no significant losses, in agreement with the statistical analysis in the fortified juice; although a slight decrease in the measured values was noted, as reported in Table 5.

### 2.6. Color

The a/b ratio of the fresh fortified tomato juice remained above 2, indicating an initially balanced red-to-yellow color profile. However, after six months of storage, a clear decline in all color parameters (L, a, and b) was observed, together with a reduction of the a/b ratio to 1,69. This marked decrease indicates a progressive loss of redness and an overall shift toward a darker and more brownish appearance.

The reduction in L, a, and b (Table 6) values suggests that both lightness and chromatic intensity were negatively affected during storage, likely due to pigment degradation and the formation of brown polymeric compounds, which are commonly associated with non-enzymatic browning reactions in fruit-based products. The presence of the polyphenolic extract may have further contributed to color instability, as phenolic oxidation products can enhance browning reactions over time.

From a technological and sensory perspective, the final a/b value of 1.69 indicates a clearly perceivable color deterioration compared to the fresh fortified product. Although the product does not appear completely degraded, the observed changes may negatively affect consumer perception, particularly in terms of visual quality and freshness perception. Therefore, while the initial formulation shows acceptable color characteristics, the results suggest that the product may require further optimization to ensure better color stability and maintain commercial acceptability over the intended shelf life.

### 2.7. Dry Matter Content

The dry residue of the non-enriched juice was 5.74 ± 0.08%, whereas the enriched juice exhibited a significantly higher value of 7.94 ± 0.10% (*p* < 0.05). The increase in dry residue observed in the enriched sample is consistent with the addition of the extract, indicating an overall increase in total solid content.

### 2.8. Nutritional Analysis of Enriched Tomato Juice

The contents of ash, sugars, and salt showed variations between the fortified juice and the control sample [35]. The increase in ash content in the fortified juice can be attributed to the ability of the solvent used to solubilize the soluble solids present in the matrix, including mineral salts and residual sugars, thus contributing to a higher overall inorganic fraction.

The observed variations in fat and protein contents were negligible and fell within the analytical uncertainty range of the measurements, suggesting that the fortification process did not substantially affect these components.

Overall, the results indicate that the extraction and addition of the polyphenolic extract do not significantly alter the lipid and protein composition of the product, while leading to a slight increase in the mineral fraction associated with co-extracted soluble solids (Table 7).

### 2.9. Sensorial Determination

**Figure 1:**
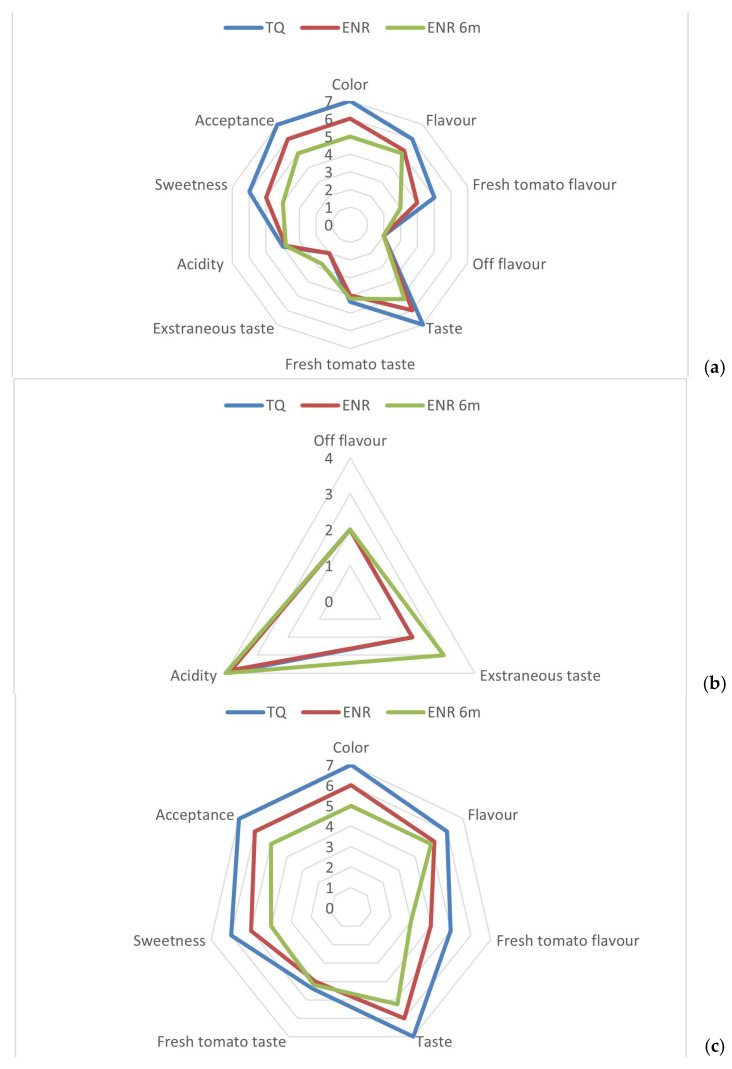
Organoleptic evaluations: comparison of the sensorial profiles of the tomato juice (TQ), fortified juice (ENR) and fortified juice after six months (ENR 6m) (**a**); sensorial profiles of the undesired features, negative descriptors (**b**); sensorial profiles of the desired features, positive descriptors (**c**).

The graphic representation of QDA (Quantitative Descriptive Analysis) obtained by processing the evaluation forms filled in by the experts is shown in Figure 1a. Taking into account the high number of data and in order to make it easier to interpret the profiles, the sensorial variable data considered negative for the relevant hybrids have been extrapolated; in particular, the data related to strange taste and flavor and to acidity have been clustered (Figure 1b). The sensory profiles of only the ‘positive’ variables are shown in Figure 1c.

The data obtained were subjected to statistical analysis, the results of which are shown in Table 8; the profiles and the statistical analysis carried out show that there are no significant differences between freshly produced tomato juice (TQ) and enriched tomato juice (ENR). After six months of storage, the enriched juice (ENR 6m) is statistically different from the tomato juice (TQ), but not significantly different from the freshly produced enriched juice (ENR); the most significant differences between juices TQ and ENR 6m are found in the color, which has darkened, in the loss of the ‘fresh tomato aroma’ component, and in the sweetness; all of this leads to a decrease in consumer acceptance.

### 2.10. Tomatine and Dehydrotomatine

In the enriched tomato juice obtained from unripe green tomatoes, tomatine and dehydrotomatine were detected at concentrations of 0.2 mg/100 g and 18.4 mg/100 g of product, respectively. The tomatine level was remarkably low and even lower than values commonly reported for fresh ripe red tomatoes, which are generally around 0.4 mg/100 g fresh weight [36]. Considering that unripe green tomatoes typically contain substantially higher concentrations of steroidal glycoalkaloids, often reaching several hundred mg/kg fresh weight, these results demonstrate a significant reduction of tomatine in the final product [37].

The marked decrease in tomatine can be attributed to the processing conditions applied, including physical treatment and thermal exposure. In addition, the pH conditions and the solvent system employed are not favorable for the solubilization of steroidal glycoalkaloids, which likely limited their transfer into the liquid phase.

Dehydrotomatine was detected at a higher concentration than tomatine (18.4 mg/100 g). This compound is a naturally occurring structural analog of tomatine and belongs to the same class of tomato glycoalkaloids. Although less extensively investigated from a toxicological perspective, available evidence suggests that the toxicological effects associated with tomato glycoalkaloids are primarily attributed to tomatine. For dehydrotomatine, toxicological data remain limited, but no evidence indicates a markedly higher toxicity, and its biological behavior is considered broadly comparable due to its structural similarity. Therefore, its occurrence should be interpreted within the context of the total glycoalkaloid content rather than as an independent toxicological risk factor [38].

Although the total content of tomatine and dehydrotomatine reached 18.6 mg/100 g in the enriched juice, this value should be interpreted considering that the product is intended as a functional ingredient for incorporation into food formulations at relatively low consumption levels. Consequently, dietary exposure to glycoalkaloids from the final food products is expected to be lower than the concentrations measured in the enriched juice. Furthermore, the marked reduction in tomatine compared with unripe green tomatoes indicates that the applied process effectively reduced the concentration of the major tomato glycoalkaloid. Nevertheless, the detected dehydrotomatine levels warrant consideration, as the total glycoalkaloid concentration is of the same order of magnitude as the acute LOAEL of 1 mg/kg body weight established by EFSA for potato glycoalkaloids (α-solanine and α-chaconine) [39]. Consumption of a 250 g serving of the enriched juice would result in an intake of approximately 46.5 mg of total glycoalkaloids, corresponding to about 0.66 mg/kg body weight for a 70 kg adult, which is below the aforementioned LOAEL. Based on this comparison, the resulting margin of exposure (MOE) would be approximately 1.5. According to Commission Recommendation (EU) 2022/561 [40], which is based on the EFSA scientific opinion on potato glycoalkaloids, a margin of exposure greater than 10 is considered indicative of no health concern. However, the toxicological relevance of this comparison remains uncertain because no health-based guidance values have been established for tomato glycoalkaloids, and the applicability of toxicological reference points derived from potato glycoalkaloids has not been fully demonstrated. Therefore, these findings should be regarded as a preliminary indication of potential dietary exposure rather than a definitive safety assessment. Further exposure studies and toxicological investigations are needed to better characterize the health implications associated with the glycoalkaloid concentrations detected in the enriched juice.

## 3. Materials and Methods

### 3.1. Materials and Reagents

Unripe green and ripe red tomato samples were collected in the Campania region (Italy) and provided by La Torrente S.r.l. (Sant’antonio Abate, Italy).

The chemicals, reagents, and materials used in this study are listed as follows. PLRP-S column 250 × 4.6 mm 5 µm particle size (Agilent Technologies, Santa Clara, CA, USA). Ferric alum ≥99% (Acros Organics, Geel, Belgium). Acetic acid glacial ≥99.8%, cresol green, ethanol 96%, methyl red, paraffin oil, sodium hydroxide (NaOH), lead acetate ≥99%, NH_4_OH 30% (Carlo Erba Reagents, Cornaredo, Italy). HPLC-grade tetrahydrofuran (Honeywell, Charlotte, NC, USA). Chlorogenic acid 94.6% (HWI Group, Rülzheim, Germany). β-Carotene ≥95%, lycopene ≥98% (Sigma-Aldrich, Merck KGaA, Darmstadt, Germany). HCl 0.1 N (PanReac AppliChem, Barcelona, Spain). 0.45 µm nylon filter membrane, Luna column 250 × 4.6 mm 5 µm particle size, Luna Omega Polar C18 column 150 × 3.0 mm 3 µm particle size, Synergi Hydro-RP column 250 × 4.6 mm 4 µm particle size, SPE, Strata SCX—strong cation-exchange (Phenomenex, Torrance, CA, USA). Dehydrotomatine ≥95%, naringenin ≥98%, rutin ≥95%, α-tomatine ≥95% (PhytoLab GmbH & Co. KG, Vestenbergsgreuth, Germany). HCl 37%, Nitric acid 65% (Scharlab, Sentmenat, Barcelona, Spain). Ascorbic acid ≥99%, caffeic acid ≥98%, catechin ≥98%, coumaric acid ≥98%, 2,6-di-tert-butyl-4-methylphenol 99% (BHT), 2,2-diphenyl-1-picrylhydrazyl (DPPH), epicatechin ≥90%, ferulic acid ≥99%, Folin–Ciocalteu reagent, gallic acid purity ≥97%, glycine standard ≥99%, oxalic acid 99%, kaempferol ≥97%, resveratrol ≥99%, sodium dihydrogen phosphate (NaH_2_PO_4_), sodium oxalate ≥99.5%, trolox ≥97%, quercetin ≥95% (Sigma-Aldrich, Merck KGaA, Darmstadt, Germany). HPLC-grade methanol, acetonitrile, TEAP 1.00 M (Supelco, Merck KGaA, Darmstadt, Germany). Boric acid, Fehling A, Fehling B, hydrogen peroxide 30%, methylene blue (1% *w*/*v* solution), sodium carbonate 99%, sulfuric acid 96% (Titolchimica, Pontecchio Polesine, Italy). Diatomaceous earth (Thermo Fisher Scientific, Waltham, MA, USA). Kjeltabs (Thomson & Capper Ltd., Runcorn, UK). Cinnamic acid USP Reference Standard (USP, Rockville, MD, USA). Acetone, ammonium thiocyanate solution (0.1 M), cyclohexane, methanol, silver nitrate solution (0.1 M) (VWR/Avantor, Radnor, PA, USA). The manuscript was prepared using Microsoft Word and Excel, specifically in its Office Professional Plus 2021 version (Microsoft Corporation, Redmond, WA, USA).

### 3.2. Polyphenols Extract

Unripe green tomatoes were processed using the semi-industrial US-juice line-ASE prototype, specifically designed for the extraction and concentration of bioactive compounds from plant matrices through the integration of ultrasound-assisted processing and accelerated solvent extraction technologies. The prototype combines crushing, ultrasound treatment, refining, concentration, drying, and solvent extraction units in a continuous and scalable process aimed at producing antioxidant-rich ingredients for enriched beverages.

Fresh tomatoes were initially vacuum-crushed using a Nilma system (Nilma S.p.A., Parma, Italy) and subsequently subjected to ultrasound-assisted treatment within the prototype processing line. Ultrasound treatment was performed using an Ecotecne ultrasound system in a dual-module configuration (model ECO 240829, TR4/2.0 line, Ecotecne Srl, Altivole, Italy). The treatment was carried out at an ultrasound frequency of 25 kHz with an acoustic power output up to 3.36 kW for 20 min under continuous-flow conditions.

Following ultrasonic treatment, the processed material was refined to remove peels and seeds and subsequently transferred to a concentration boule. The obtained concentrate reached approximately 18° Brix soluble solids content.

The concentrate was subsequently dried using a Sandvik dryer (Sandvik Process Systems, Sandviken, Sweden) for 4 h at temperatures below 60 °C in order to obtain a stable matrix suitable for pressurized solvent extraction.

The dried material was then subjected to accelerated solvent extraction using the ASE EXTREVA system (Thermo Fisher Scientific Inc., Waltham, MA, USA) [41]. Extraction was carried out using ethanol/water (50:50, *v*/*v*) as the extraction solvent, with a sample-to-solvent ratio of 1:20 (*w*/*v*). Operating conditions were set at 55 °C and 28 ± 3 bar. The extraction process consisted of one static cycle with a duration of 15 min.

At the end of the extraction process, the solvent was removed under reduced pressure, and the resulting polyphenolic extract was concentrated approximately 10-fold. The final extract showed a total polyphenol content of 1788.89 mg GAE/100 g. Extraction yield, calculated on a dry weight basis, corresponded to 84.8%.

### 3.3. Fortified Tomato Juice

The obtained polyphenolic extract was added to red tomato juice immediately prior to packaging and thermal pasteurization. Specifically, 152 g of extract were incorporated into 5 L of tomato juice, corresponding to a final extract concentration of 30.4 g/L in the fortified beverage. The added extract contributed approximately 2.3% of total dry matter to the final formulation.

Fortification was carried out under continuous mechanical stirring at 400 rpm for 15 min at 90 ± 1 °C in order to ensure homogeneous distribution of the extract within the juice matrix. The fortified juice was then packaged in glass jars and subjected to thermal pasteurization at 99 ± 1 °C in a thermostatically controlled water bath for 25 min, ensuring microbiological safety while minimizing the degradation of quality attributes and bioactive compounds, including vitamin C, polyphenols, and organoleptic properties.

Each production batch consisted of 5 L of fortified tomato juice.

The fortification process was performed on two independent batches, and all analytical determinations were carried out in triplicate to evaluate process reproducibility and product stability.

The thermally stabilized fortified juice was packaged in 106 mL glass jars sealed with twist-off screw-cap closures, leaving a headspace of approximately 13 mm. A total of 40 jars were stored at room temperature (22 ± 5 °C) under natural light exposure conditions. Samples were analyzed immediately after production and after 6 months of storage in order to evaluate the stability of physicochemical and functional properties during shelf life. Furthermore, microbiological analyses were performed at the end of the storage period to assess product safety and microbiological stability.

### 3.4. Total Polyphenols

The total phenolic content (TPC) has been determined using the Folin–Ciocalteu method [42], following a slightly modified procedure. Briefly, an aliquot of the extract (20–100 μL) has been mixed with 1000 μL of diluted Folin–Ciocalteu reagent (1:5 *v*/*v* with distilled water), and 800 μL of sodium carbonate solution (20%, *w*/*v*) has been added to the mixture.

The reaction mixture has been incubated in the dark at room temperature for 120 min. The absorbance has been then measured at 760 nm using a UV–VIS spectrophotometer (UV-1900i, Shimadzu Corporation, Kyoto, Japan) against a reagent blank.

A calibration curve has been prepared using gallic acid as the reference standard (50–300 mg L^−1^; R^2^ ≥ 0.99). The results have been expressed as milligrams of gallic acid equivalents for 100 g of sample (mg GAE 100 g^−1^ of FW).

All analyses carried out in this study have been performed in triplicate, and results have been reported as mean ± standard deviation.

### 3.5. DPPH Radical Scavenging Activity

The antioxidant activity of the extracts has been evaluated using the DPPH assay based on the scavenging of the stable radical 2,2-diphenyl-1-picrylhydrazyl, according to a previously described method with slight modifications [43].

Briefly, 50 μL of the sample extract at different concentrations has been mixed with 2450 µL of a methanolic or acetonic DPPH solution (0.125 mM). The reaction mixture has been incubated in the dark at room temperature for 30 min.

The decrease in absorbance has been measured at 517 nm using a UV–VIS spectrophotometer (UV-1900i, Shimadzu Corporation, Kyoto, Japan) against a methanol or acetone blank. The radical scavenging activity has been calculated as a percentage inhibition using the following equation:

$$\mathrm{DPPH}\;\mathrm{scavenging}\;\mathrm{activity}\;(\%)\;=\;\frac{A_{0}-A_{s}}{A_{0}}\text{×}\;100$$

where A_0_ is the absorbance of the control and A_s_ is the absorbance of the sample.

### 3.6. Identification and Quantification of Phenolic Compounds by HPLC-DAD

The extraction method has been taken according to Moilanen et al. [44] with a minor modification. Firstly, 1 g of enriched tomato juice or concentrated extract and 2 mL of methanol have been added to a 5 mL centrifuge tube, and extracted at room temperature for 1 h. The extracting solution has been centrifuged for 4 min, and the supernatant has been filtered using a 0.45 µm nylon membrane.

Polyphenol speciation has been carried out using high-performance liquid chromatography coupled with a diode array detector (HPLC Jasco-DAD MD-2018 Plus, Jasco, Tokyo, Japan) [45]. The chromatographic analysis has been performed on a reversed-phase C18 column (Luna 250 × 4.6 mm, 5 µm particle size) maintained at 30 °C.

The mobile phase consisted of solvent A (methanol) and solvent B (1% acetic acid in water). The following gradient elution program has been applied: 0–30 min, 5–30% A; 30–40 min, 30–40% A; 40–45 min, 40–50% A; 45–50 min, 50–70% A; 50–55 min, 70–40% A; 55–57 min, 40–5% A; 57–60 min, 5% A by re-equilibration to initial conditions. The flow rate has been set at 1.1 mL/min, and the injection volume has been 10 µL.

Detection has been performed at multiple wavelengths: 240 nm, 280 nm and 322 nm.

Identification of individual polyphenols has been achieved by comparing retention times and UV–VIS spectra with those of authentic standards, including Caffeic acid, Catechin, Chlorogenic acid, Cinnamic acid, Coumaric acid, Epicatechin, Ferulic acid, Kaempferol, Naringenin, Quercetin, Rutin, and Resveratrol.

The analytical method was validated following standard analytical performance criteria. Limits of detection (LOD) and quantification (LOQ) were estimated at 0.05 mg/kg and 0.10 mg/kg, respectively.

The calibration curve was constructed over a concentration range between 0.5 and 10 mg/kg and demonstrated excellent linearity (R^2^ > 0.999).

Accuracy was assessed through recovery experiments performed at three spiking levels (low, medium, and high), yielding recoveries between 85% and 105%. Precision was evaluated as repeatability, expressed as relative standard deviation (RSD), which remained below 5% for all analytes.

Matrix effects were evaluated by comparing solvent-based and matrix-matched calibration curves, and no significant differences in slope were observed; therefore, external calibration was considered suitable for quantification.

Compound identification was confirmed by combining retention time data with UV–Vis spectral information obtained by diode array detection (DAD), in addition to comparison with authentic reference standards.

### 3.7. Determination of Vitamin C

Vitamin C content has been determined by high-performance liquid chromatography [46].

Briefly, 5 g of the sample has been extracted with 50 mL of 1% (*w*/*v*) oxalic acid solution to stabilize ascorbic acid and prevent oxidation. The mixture has been vortexed and filtered through a 0.45 µm nylon membrane prior to analysis.

Chromatographic analysis has been carried out using an HPLC system equipped with a UV detector (HPLC Jasco-DAD MD-2018 Plus, Jasco, Tokyo, Japan). Separation has been performed on PLRP-S (250 × 4.6 mm, 5 µm particle size) at 35 °C.

The mobile phase consisted of 0.2 M Na_2_H_2_PO_4_ in water, eluted at a flow rate of 0.6 mL/min.

The injection volume has been 20 µL.

Detection has been performed at 240 nm. Identification and quantification have been achieved by comparison with an external standard of ascorbic acid, using a calibration curve (R^2^ > 0.999).

Results have been expressed as mg of vitamin C per 100 g dry weight.

### 3.8. Extraction of Carotenoids by Accelerated Solvent Extraction (ASE) and HPLC-DAD Analysis of Lycopene

Carotenoids have been extracted using an accelerated solvent extraction system (ASE EXTREVA, Thermo Fisher Scientific Inc., Waltham, MA, USA) [47]. Approximately 2 g of homogenized sample has been mixed with 0.5 g of NaCl, 1 mL of paraffin oil, and diatomaceous earth and loaded into stainless steel extraction cells (10 mL).

Extraction has been performed using tetrahydrofuran (THF) containing 0.1% 2,6-di-tert-butyl-4-methylphenol (BHT) to prevent oxidation. The extraction conditions have been as follows: temperature 55 °C, extraction time 5 min, flow rate 1 mL/min, cell fill volume 50%, gas flow rate 10 mL/min, nitrogen purge time 30 s.

The combined extracts have been collected in amber glass flasks, made up to volume and then filtered using a 0.45 µm nylon membrane prior to chromatographic analysis.

Lycopene has been extracted and quantified using high-performance liquid chromatography (HPLC-DAD, Waters Corporation, Milford, MA, USA) [48].

Chromatographic separation has been performed on a Synergi Hydro-RP (250 × 4.6 mm, 4 µm particle size) at 35 °C. The mobile phase consisted of a methanol:THF:water mixture (57:37:6, *v*/*v*/*v*) at a flow rate of 1.2 mL/min. The injection volume has been 10 µL. Detection has been carried out at 450 nm.

Identification has been achieved by comparing retention times and UV–VIS spectra with authentic standards of lycopene and β-carotene.

Quantification has been performed using external calibration curves (R^2^ > 0.999), and results have been expressed as mg/kg of dry weight (DW).

All analyses have been performed under dim light conditions to minimize carotenoid degradation.

### 3.9. Color

Color measurements have been performed using a colorimeter (LabScan XE, HunterLab, Reston, VA, USA). The color parameters have been expressed in the Hunter Lab color space, where L indicates lightness, a represents the red/green coordinate, and b the yellow/blue coordinate. Measurements have been carried out under standard illuminant conditions (D65, 10° observer) and calibrated using a white standard tile [49].

### 3.10. Nutritional Analysis of Enriched Tomato Juice

The nutritional composition of fortified tomato juice has been determined by standard analytical methods.

Moisture content has been determined by drying the samples under vacuum at 70 °C for 4 h [50].

Ash content has been measured by incineration in a muffle furnace at 550 °C for 4 h [50].

Protein content has been determined using the Kjeldahl method, and total nitrogen has been converted to protein using a factor of 6.25 [51].

Total lipids have been determined by microwave extraction using cyclohexane as solvent [52].

Total carbohydrate content has been calculated by difference, subtracting the sum of moisture, protein, lipids, and ash from 100. Results have been expressed as g/100 mL. Reducing sugars have been determined by Fehling’s titration method [50].

The sodium chloride (NaCl) content of fortified tomato juice has been determined by argentometric titration using the Mohr method [50].

### 3.11. Sensorial Determination

Sensory and organoleptic evaluations have been carried out by a tasting panel composed of ten expert evaluators, selected based on their previous experience in the sensory analysis of tomato-derived products and subjected to a preliminary training session to align the descriptors and evaluation methods.

The assessments were carried out in two separate sessions; in the first, the tomato juice was tasted both in its natural state and after enrichment; in the second, the enriched juice was assessed after six months of storage at 22 ± 5 °C.

Samples, identified using a random numerical code to ensure anonymity, have been presented under neutral lighting conditions (4000 K) and served at a temperature of 25 ± 2 °C; water was provided between evaluations to cleanse the palate.

Each evaluator assessed the samples under, and their judgments have been recorded using a questionnaire comprising 10 sensory variables. Among these, four have been considered primary variables, while the remaining ones have been treated as secondary descriptors providing further detail.

For the primary variables, which consist of color, flavor, taste and acceptance, scores ranged from zero (extremely unpleasant) to ten (extremely pleasant). In particular, for color evaluation, a score of zero corresponded to a brick-red hue, while a score of ten corresponded to a bright red color.

The secondary variables consist of: fresh tomato flavor, off flavor, fresh tomato taste, extraneous taste, acidity and sweetness. For their evaluation form, the following perception levels have been considered, with corresponding scores in parentheses: absence (2), slight presence (4), moderate presence (6), and strong presence (8). These scores have been used to calculate mean values.

### 3.12. Tomatine and Dehydrotomatine

The determination of glycoalkaloids was performed using an accelerated solvent extraction (ASE) approach.

Briefly, 1 g of dried sample matrix was mixed with diatomaceous earth and loaded into the extraction cell, which was subsequently placed in the ASE system (Extreva, Thermo Fisher Scientific Inc., Waltham, MA, USA) for solvent extraction. The operating conditions were optimized to improve process efficiency and sustainability. The extraction solvent consisted of an H_2_O:EtOH mixture (30:70, *v*/*v*) acidified to pH 3.2. Extractions were performed at 55 °C.

After extraction, the pH of the extract was adjusted using 30% NH_4_OH, and the solution was stored at 4 °C for 12–24 h [53]. The following day, samples were centrifuged for 30 min at −4 °C and 9500 rpm. The precipitate was recovered and resuspended in 2 mL of methanol.

Samples were then purified by solid-phase extraction (SPE, Strata SCX—strong cation-exchange). The cartridge was conditioned with 3 mL of methanol, followed by 3 mL of 5% acetic acid and 6 mL of 5% methanol. After sample loading, interfering compounds were removed, while target analytes were eluted using 6 mL of 2.5% NH_4_OH. The eluate was then concentrated to dryness using a rotary evaporator, reconstituted in 2 mL of methanol, filtered through a 0.45 µm nylon membrane, and transferred into a 2 mL vial for HPLC-DAD analysis (HPLC Jasco-DAD MD-2018 Plus, Jasco, Tokyo, Japan).

Chromatographic analyses were performed using an HPLC system equipped with a Luna Omega Polar C18 column (150 × 3.0 mm, 3 µm particle size). The mobile phase consisted of acetonitrile (A) and 25 mM TEAP buffer (B), at a flow rate of 0.4 mL/min.

The gradient elution program was as follows: 20:80 (A/B) initially; 45:55 (A/B) from 0 to 12 min; 55:45 (A/B) from 12 to 17 min; 57:43 (A/B) from 17 to 20 min; and 20:80 (A/B) from 20 to 30 min.

### 3.13. Statistics

All experiments were performed using two independent replicates, each derived from separate and independently prepared samples. For each replicate, measurements were performed in technical triplicate to ensure analytical repeatability. Technical replicates were averaged prior to statistical analysis to avoid pseudo-replication.

Data are expressed as mean ± standard deviation (SD). Where appropriate, 95% confidence intervals were also calculated to provide additional information on data dispersion and estimation precision.

Statistical analyses were performed using one-way or two-way analysis of variance (ANOVA). When significant differences were detected, Tukey’s honestly significant difference (HSD) post hoc test was applied to compare means. Statistical significance was set at *p* < 0.05.

Prior to conducting ANOVA, data were assessed for compliance with model assumptions. Normality of residuals was verified using the Shapiro–Wilk test.

When the assumption of normality has not been met, the Kruskal–Wallis test has been applied, followed by Dunn’s multiple comparison test. Statistical significance has been set at *p* < 0.05.

Data have been processed using Microsoft Excel 2021 (Microsoft Corp., Redmond, WA, USA). Statistical analysis has been carried out using Jamovi cloud (version 2.7.27).

## 4. Conclusions

A study was conducted on the development of an enriched tomato juice enriched with natural bioactive extracts obtained from unripe green tomatoes. The enrichment process successfully increased the total polyphenol content of the final product and modified its polyphenolic profile, confirming the effective transfer of bioactive compounds into the tomato juice matrix.

Although the fortified juice initially exhibited enhanced antioxidant activity compared to the control sample, the stability of several quality parameters was affected during storage. In particular, a marked decrease in DPPH radical scavenging activity was observed after 6 months, especially in the methanolic fraction, suggesting a substantial reduction in hydrophilic antioxidant capacity over time. Similarly, color parameters progressively declined during storage, with a reduction in the a/b ratio indicating noticeable browning and loss of the characteristic red color of tomato juice.

Moreover, while lycopene content remained relatively stable, vitamin C and some antioxidant-related properties showed reductions following processing and storage, indicating that the technological treatment and storage conditions influenced the stability of sensitive bioactive compounds. Sensory evaluation also suggested a decrease in product acceptability after storage, particularly in relation to color and overall appearance.

Overall, the results demonstrate the feasibility of enriching tomato juice with polyphenol-rich extracts derived from unripe green tomatoes; however, the study also highlights important limitations related to antioxidant stability, color preservation, and sensory quality during storage. Therefore, further optimization of processing conditions, packaging systems, oxygen control, and storage parameters is required before considering potential functional, nutraceutical, or commercial applications of the proposed beverage.

## Figures and Tables

**Table 1:** Total polyphenol content (mg GAE 100 g^−1^ fresh weight (FW)) in tomato juice (TQ), fortified tomato juice (ENR), and fortified tomato juice after six months (ENR 6m).

Samples	Total Polyphenols
TQ	40.97 ± 1.08 a
ENR	82.45 ± 0.51 b
ENR 6m	71.44 ± 0.93 c

Different letters indicate statistically significant differences (*p* < 0.05, Tukey’s HSD test).

**Table 2:** DPPH radical scavenging activity (% inhibition) in tomato juice (TQ), fortified tomato juice (ENR), and fortified tomato juice after six months (ENR 6m).

Samples	DPPH Activity (Methanol Extract)	DPPH Activity (Acetone Extract)
TQ	37.85 ± 1.62 a	23.02 ± 2.83 a
ENR	46.08 ± 1.20 b	45.41 ± 1.53 b
ENR 6m	35.50 ± 1.58 a	34.02 ± 1.17 c

Different letters within the same column indicate statistically significant differences (*p* < 0.05, Tukey’s HSD test).

**Table 3:** Polyphenolic profile (mg/kg dry weight (DW)) in tomato juice (TQ), fortified tomato juice (ENR), and fortified tomato juice after six months (ENR 6m).

Compound	TQ	ENR	ENR 6m
Caffeic Acid	25.72 ± 0.10 a	53.61 ± 4.60 b	57.64 ± 2.20 b
Cinnamic Acid	<0.10	<0.10	<0.10
Chlorogenic Acid	147.97 ± 2.21 a	103.95 ± 6.92 b	98.99 ± 3.96 b
Coumaric Acid	1.57 ± 0.17 a	11.00 ± 1.97 b	9.15 ± 0.51 b
Ferulic Acid	0.81 ± 0.10 a	10.96 ± 1.69 b	<0.10
Catechin	43.97 ± 3.41	<0.10	<0.10
Epicatechin	55.80 ± 8.27 a	21.08 ± 1.47 b	23.01 ± 2.36 b
Kaempferol	64.58 ± 1.210 a	268.77 ± 2.48 b	262.49 ± 4.92 b
Naringenin	<0.10	50.97 ± 0.95 a	35.19 ± 4.85 b
Resveratrol	1.74 ± 0.17	<0.10	<0.10
Rutin	41.34 ± 6.35 a	62.26 ± 3.14 b	58.02 ± 5.57 b

Different letters within the same lines indicate statistically significant differences (*p* < 0.05, Tukey’s HSD test).

**Table 4:** Vitamin C content (mg 100 g^−1^ dry weight (DW)) in tomato juice (TQ), fortified tomato juice (ENR), and fortified tomato juice after six months (ENR 6m).

Samples	Vitamin C
TQ	61.01 ± 5.01 a
ENR	32.12 ± 1.39 b
ENR 6m	30.19 ± 3.60 b

Different letters within the same column indicate statistically significant differences (*p* < 0.05, Tukey’s HSD test).

**Table 5:** Lycopene content (mg kg^−1^ dry weight (DW)) in tomato juice (TQ), fortified tomato juice (ENR), and fortified tomato juice after six months (ENR 6m).

Samples	Lycopene
TQ	4934.93 ± 149.83 a
ENR	4822.39 ± 46.42 a
ENR 6m	4618.42 ± 52.39 a

Different letters indicate statistically significant differences (Kruskal–Wallis test followed by Dunn’s post hoc test, *p* < 0.05).

**Table 6:** Color parameters of tomato juice (TQ), fortified tomato juice (ENR), and fortified tomato juice after six months (ENR 6m).

Samples	L	a	b	a/b
TQ	25.73 ± 0.01 a	33.75 ± 0.04 a	14.83 ± 0.01 a	2.28 a
ENR	25.41 ± 0.03 a	30.25 ± 0.01 a	14.08 ± 0.03 a	2.15 a
ENR 6m	19.66 ± 0.01 b	18.17 ± 0.01 b	10.74 ± 0.02 b	1.69 b

Different letters indicate statistically significant differences in the same column (Kruskal–Wallis test followed by Dunn’s post hoc test, *p* < 0.05).

**Table 7:** Nutritional composition (g 100 g^−1^) of tomato juice (TQ) and fortified tomato juice (ENR).

Samples	Ash	Proteins	Fat	Sugar	Salt
TQ	0.65 ± 0.01 a	1.10 ± 0.21 a	0.12 ± 0.05 a	3.14 ± 0.05 a	0.06 ± 0.01 a
ENR	0.84 ± 0.01 b	1.54 ± 0.30 a	0.16 ± 0.04 a	3.31 ± 0.04 b	0.09 ± 0.01 b

Different letters indicate statistically significant differences in the same column (Kruskal–Wallis test followed by Dunn’s post hoc test, *p* < 0.05).

**Table 8:** Organoleptic characteristics of the produced tomato juices: mean values of the sensory evaluation performed by expert panelists for tomato juice (TQ), fortified tomato juice (ENR), and fortified tomato juice after six months of storage (ENR 6m).

Samples	Color	Flavor	Fresh Tomato Flavor	Off Flavor	Taste	Fresh Tomato Taste	Extraneous Taste	Acidity	Sweetness	Acceptance
TQ	7 a	6 a	5 a	2 a	7 a	4 ab	2 a	4 a	6 a	7 a
ENR	6 ab	6 a	5 ab	2 a	6 a	4 a	2 a	4 a	5 a	6 ab
ENR 6m	5 b	5 a	3 b	2 a	6 b	4 b	2 b	4 a	4 a	5 b

Different letters indicate statistically significant differences in the same column (Kruskal–Wallis test followed by Dunn’s post hoc test, *p* < 0.05).

## Data Availability

The data presented in this study are available on request from the corresponding author.
